# Chilling- and Freezing- Induced Alterations in Cytosine Methylation and Its Association with the Cold Tolerance of an Alpine Subnival Plant, *Chorispora bungeana*

**DOI:** 10.1371/journal.pone.0135485

**Published:** 2015-08-13

**Authors:** Yuan Song, Lijun Liu, Yanhao Feng, Yunzhu Wei, Xiule Yue, Wenliang He, Hua Zhang, Lizhe An

**Affiliations:** 1 Ministry of Education Key Laboratory of Cell Activities and Stress Adaptations, School of Life Sciences, Lanzhou University, Lanzhou, China; 2 Department of Biology, University of Konstanz, Universitätsstrasse 10, Konstanz, Germany; South China Agricultural University, CHINA

## Abstract

Chilling (0–18°C) and freezing (<0°C) are two distinct types of cold stresses. Epigenetic regulation can play an important role in plant adaptation to abiotic stresses. However, it is not yet clear whether and how epigenetic modification (i.e., DNA methylation) mediates the adaptation to cold stresses in nature (e.g., in alpine regions). Especially, whether the adaptation to chilling and freezing is involved in differential epigenetic regulations in plants is largely unknown. *Chorispora bungeana* is an alpine subnival plant that is distributed in the freeze-thaw tundra in Asia, where chilling and freezing frequently fluctuate daily (24 h). To disentangle how *C*. *bungeana* copes with these intricate cold stresses through epigenetic modifications, plants of *C*. *bungeana* were treated at 4°C (chilling) and -4°C (freezing) over five periods of time (0–24 h). Methylation-sensitive amplified fragment-length polymorphism markers were used to investigate the variation in DNA methylation of *C*. *bungeana* in response to chilling and freezing. It was found that the alterations in DNA methylation of *C*. *bungeana* largely occurred over the period of chilling and freezing. Moreover, chilling and freezing appeared to gradually induce distinct DNA methylation variations, as the treatment went on (e.g., after 12 h). Forty-three cold-induced polymorphic fragments were randomly selected and further analyzed, and three of the cloned fragments were homologous to genes encoding alcohol dehydrogenase, UDP-glucosyltransferase and polygalacturonase-inhibiting protein. These candidate genes verified the existence of different expressive patterns between chilling and freezing. Our results showed that *C*. *bungeana* responded to cold stresses rapidly through the alterations of DNA methylation, and that chilling and freezing induced different DNA methylation changes. Therefore, we conclude that epigenetic modifications can potentially serve as a rapid and flexible mechanism for *C*. *bungeana* to adapt to the intricate cold stresses in the alpine areas.

## Introduction

Epigenetic modifications (e.g., DNA methylation) are ubiquitous mechanisms that can bring about heritable phenotypic changes through the regulation of gene expression without changing DNA sequences [1–4]. A number of recent studies argue that DNA methylation/demethylation is involved in regulating the transcriptional activities of stress-response genes in plants [5–7]. Furthermore, this stress-induced DNA methylation change can be carried forward as within-generation and/or transgenerational epigenetic memories (i.e., soft inheritance) in plants to effectively cope with subsequent environmental stresses [8–11]. Epigenetic regulation and the soft inheritance thereof have been proposed as potential driving forces with respect to evolutionary changes over time (e.g., in natural selection) [12–16].

In nature, cold is a major environmental abiotic stress that adversely affects plant growth and survival, and thereby constrains the geographical distribution of plants and agricultural productivity [17]. Cold stresses consist of chilling (0–18°C) and freezing (<0°C), which are related to different inhibition processes in plant tissues [18, 19]. For instance, chilling can induce the inhibition of water uptake in plants, while freezing can cause cellular dehydration due to extracellular ice formation [17]. Indeed, the modification of DNA methylation (i.e., methylation and demethylation) as a relatively rapid epigenetic regulator can potentially provide more-flexible genomic parameters for plants responding to various cold stresses [20–22]. For instance, the adaptive ability of *Arabidopsis* and maize to cold stress is associated with epigenetic variations that can effectively “sense” the changed ambient temperature [23–25]. While a mounting number of studies have argued that precise molecular regulations are supposedly involved in the response and adaptation of plants to cold stress [26–29], the underlying rules of epigenetic variation during this process hitherto have remained less clear. In particular, whether and to what extent the response and adaptation to the chilling (0–18°C) and freezing (<0°C) are involved in differential epigenetic regulations (i.e., changes in DNA methylation) are largely unknown. Methylation-sensitive amplified fragment-length polymorphism (MS-AFLP) has been widely used to study the global DNA methylation status of a plant species, especially those lacking sequenced genomes [30–32].

**Fig 1:**
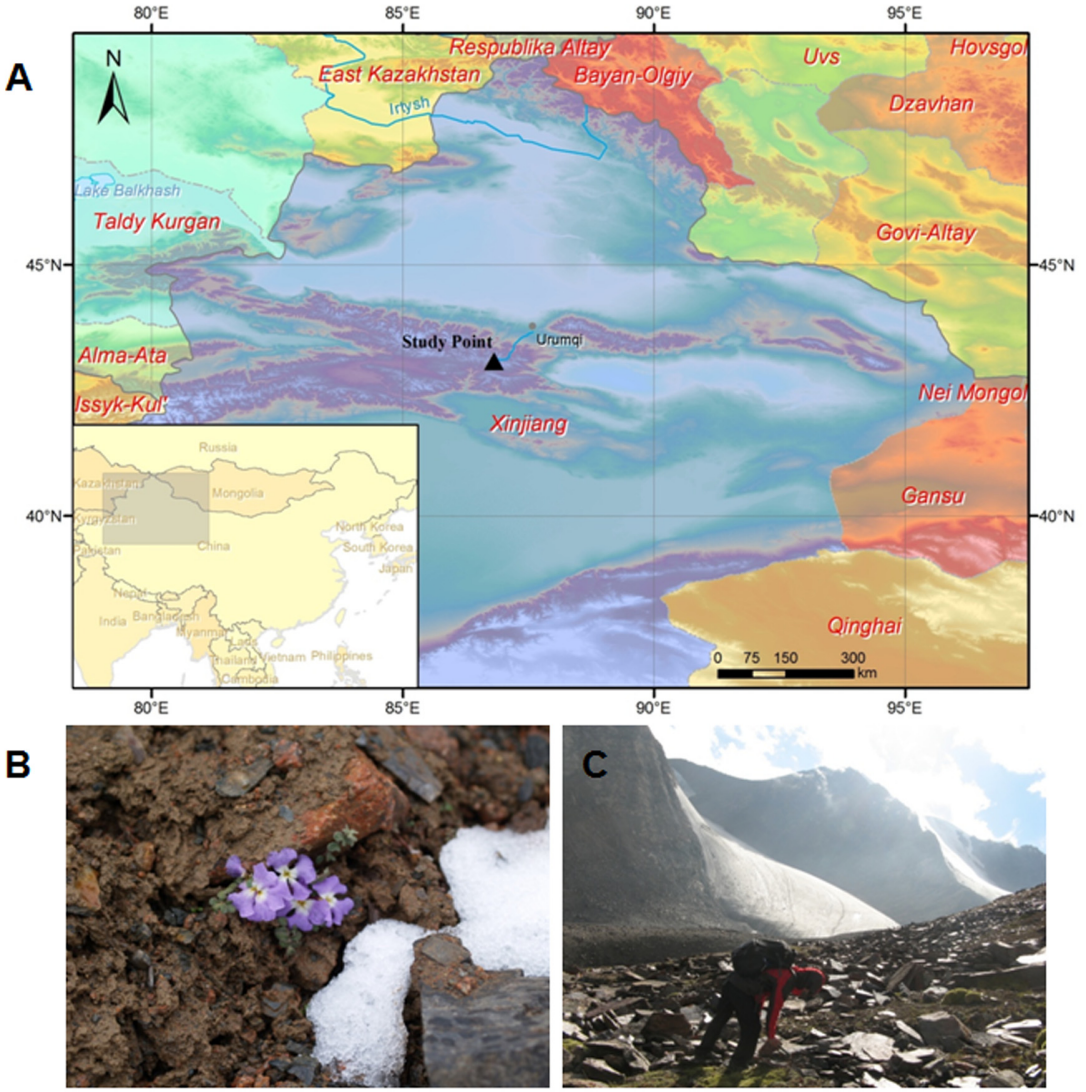
The plant material collection site and a photograph of *C*. *bungeana*. (A) The black-filled triangle displays the geographic position of the collection site on the map, approximately 43°05’N, 86°49’E, altitude 3,800–3,900 m, near the No. 1 glacier in the source area of the Urumqi River in the Tianshan Mountains, Xinjiang, China. (B) Photograph of *C*. *bungeana*, flowering in snow and ice. (C) A researcher was working in the alpine talus of the collection site.

*Chorispora bungeana* Fisch. & C.A. Mey is a perennial alpine subnival plant species that naturally grows and survives in the alpine regions (2000–4200 m) in Asia [33, 34]. As previous studies have reported, *C*. *bungeana* is naturally distributed in freeze-thaw tundra in the origin of the Urumqi River in the Tianshan Mountains, China (Fig 1) [35]. In this region, snow and hail often occur during the favorable growing season (from June to September) of *C*. *bungeana*. As a consequence, the ambient air temperature frequently fluctuates from 20°C (chilling) to -10°C (freezing) during the growing season. In addition, fluctuations between chilling and freezing also often occur within a day (i.e., 24 h) in this region [35]. *C*. *bungeanais* belongs to the Chorispora genus, Cruciferae family, and it exhibits strong tolerance to multiple abiotic stresses, especially cold stress. Long-term field studies and controlled greenhouse growth experiments have revealed that *C*. *bungeana* does not possess special morphological structure to withstand harsh habitat, but instead contains large amounts of free fatty acids, neutral amino acid, soluble sugar, Mg2+-ATPase activity, and unsaturated fatty acids *et*.*al*. [34, 36–40], suggesting that this plant has a physiological metabolic resistance mechanism. Therefore, we think *C*. *bungeana* is an ideal organism to isolate and clone antifreeze genes for heterologous expression and application. Because plant survival is closely dependent on environmental growth conditions, epigenetic research is required.

In this study, we set out to investigate the DNA methylation change patterns of *C*. *bungeana* in response to chilling and freezing stresses using the MS-AFLP technique and integrated statistical analyses. Plants of *C*. *bungeana* were treated at 4°C (chilling) and -4°C (freezing), thereby allowing us to disentangle the dynamic response of DNA methylation variations to the two types of cold stresses. Furthermore, forty-three cold-induced polymorphic DNA methylation bands randomly selected above were cloned and sequenced. Among these bands, three of the cloned fragments were homologous to genes encoding alcohol dehydrogenase (*CbADH1*), UDP-glucosyltransferases (*CbUGT*), and polygalacturonase-inhibiting protein (*CbPGIP*). In addition, the level of 5-methylcytosine binding at the three genes and their mRNA expression were tested by chromatin immunoprecipitation assays (ChIP) and quantitative real-time PCR (qPCR) analysis.

Our results show alterations in cytosine methylation induced by chilling and freezing in *C*. *bungeana*, indicating that epigenetic modification (i.e., DNA methylation) can potentially serve as a rapid and flexible adaptive mechanism for *C*. *bungeana* to deal with intricate cold stresses in nature.

## Materials and Methods

### Plant material and cold treatments

Whole plants of *C*. *bungeana* together with their growing soils were collected from a cirque in the freeze—thaw tundra where the species naturally occurs (with a 3,800–3,900 m height above sea level). The field studies did not involve endangered or protected species, and no specific permissions were required for our activities in the location of our study (e.g., GPS coordinates).

Plant regeneration of *C*. *bungeana* via embryonic calli was performed as described by Wang *et al*, (2006) [41] and Chang *et al*. (2005) [34] with modifications. The cotyledons were cut into 10-mm segments using an aseptic operation knife. The segments were then cultured on (callus-inductive) MS basal medium containing 1 mg L^-1^ 2,4-dichlorophenoxyacetic acid (2,4-D), 0.2 mg L^-1^ 6-Benzylaminopurine (6-BA), 3% sucrose and 0.8% agar, pH 5.8, for approximately three weeks. Then, plant seedlings were regenerated from the calluses on MS basal medium that was supplemented with 0.5 mg L^-1^ 6-BA, 3% sucrose and 0.6% agar for approximately four weeks. The regenerated seedlings were then transferred to new MS medium containing 3% sucrose in the plant culture flasks and grown at 23°C under a photoperiod (16 h: 8 h, light: dark) and 60% humidity for approximately three weeks before treatments.

When the regenerated plants from the subculture were approximately 5 cm tall, they were used for experiments. For low temperature treatments, the regenerated plants of *C*. *bungeana* were treated in the low-temp incubator (SANYO, MIR-254, Japan). The three regenerated plants were randomly selected and harvested in each of the chilling (4°C), freezing (-4°C) and control treatments (23°C) 0, 0.5, 3, 12 and 24 h after the cold treatment initiated. All of the collected plant samples were immediately frozen in liquid nitrogen and then stored at -80°C for further analysis.

### MS-AFLP analysis

Total DNA was extracted from the three plants that were randomly selected and harvested per treatment, based on the protocol using the Hexadecyl-trimethyl-ammonium-bromide (CTAB) as described in [42] with some modifications. The DNA was then re-suspended in 50 μl of sterile water. The quality of the extracted DNA was checked using the NanoDrop Spectrometer (ND-1000 Spectrophotometer, PeQLab).

In order to assess epigenetic variations of *C*. *bungeana* in response to the chilling and freezing stresses, a digestion-ligation system *Eco*RI/*Hpa*II was built [43]. For restriction-digested reactions, 400 ng of total genomic DNA was endonuclease-digested with 3 U of *Eco*R*I*, 1× RT buffer (0.01 mol L^-1^ Tris-HCl, 0.01 mol L^-1^ MgAc2, 0.05 mol L^-1^ KAc) for 5 h at 37°C. The digested products were incubated overnight with 4 U of *Hpa*II (10 U *Hpa*II per microgramme DNA). The reaction was terminated by a 65°C water bath for 15 min. Then, 50 pmol of *Eco*RI and *Hpa*II double-stranded adapters (S1 Table) was ligated to the restriction products with 1.5 U of T4 DNA ligase (New England Biolabs), 1× RT buffer (1 mmol ATP) overnight at 16°C in a final volume of 20 μl. A pre-selective amplification system was conducted in a 25-μl reaction using 1 μl of ligated product as a template for PCR amplification with the following primers: 5’-GACTGCGTACCAATTC-3’ and 5’-GATGAGTCCTGAGCGG-3’. The reactions were carried out in 21 cycles at 94°C for 30 s, 56°C for 1 min and 72°C for 1 min with a 10-min final extension. The products were diluted 20-fold, and then 2 μl was used for the selective amplification. For the selective primers, we added three selective nucleases at the 3’ end of pre-selective primers, such that the abundant polymorphism can be detected. The same 25-μl reactions contained 30 ng of the following selective primers pairwise: *Eco*RI+AAC, *Eco*RI+ACC, *Eco*RI+ACT, *Eco*RI+ATG and *Eco*RI+CAT combined with *Hpa*II+AGA, *Hpa*II+AGC and *Hpa*II+AGG, 0.2 mM dNTPs, 10× PCR buffer and 2 U of Taq polymerase (TaKaRa). The touchdown reaction program was: 94°C for 30 s, 65°C for 30 s and 72°C for 1 min with the annealing temperature decreased by 0.7°C per cycle over 13 cycles. The remaining 23 cycles were 94°C for 30 s, 56°C for 30 s, and 72°C for 1 min with a 10-min final extension. The final amplification products were denatured at 94°C for 10 min and separated by a 6% denaturing polyacrylamide gel with 7.5 M urea. The gels were stained with 0.1% silver nitrate solution (0.5% formaldehyde) for 30 min. The fragment scores were estimated by Quantity one 1-D Analysis software (Bio-Rad, Hercules, USA) after photo-documented staining development.

Cold-induced polymorphic DNA methylation bands were randomly detected. The target gel fragments were purified by the Poly-Gel DNA Extraction Kit (Omega, USA), and 2 μl of eluted DNA was used for PCR amplification in a final volume of 50 μl with the same reaction conditions as in the pre-amplification described above. The amplified DNA fragments were purified, inserted into pEASY-T3 cloning vector, and transfected into DH5α competent *E*. *coli* cells. The acquired positive clones were tested and sequenced. The potential function of the sequences was analyzed by TAIR BLAST (http://www.arabidopsis.org/).

### Data analysis

Analyses of the MS-AFLP results were based on fragment presence (1) / absence (0) matrices for individual samples as obtained with the fragment amplification of *Eco*RI and *Hpa*II primer combinations.

A generalized linear model (GLM) was used for MS-AFLP data to assess epigenetic variations in response to the chilling (4°C) and freezing (-4°C) treatments at different time points (0.5, 3, 12 and 24 h). In the model, dummy indicator variables were manually constructed for eight cold treatments [44]. The difference between each of the eight cold treatments and 23°C was assessed by comparing the full model with the model from which the respective term was removed using the Generalized likelihood ratio test (GLRT). In order to account for the effects of the markers and treatment time, we also included these effects as explanatory variables in the GLM models and examined the significance by the GLRT. In order to check and compare the results of the GLM models, similar analyses were also executed by a *G* test. Patterns of DNA methylation changes (i.e., methylation, demethylation and NA) were also compared among the room temperature 23°C, chilling (4°C) and freezing (-4°C), treatments using *G* test and Fisher's exact test. NA indicates that the status of the DNA methylation of the loci was unknown. Moreover, a general comparison for the relative proportion of methylation and demethylation (i.e., without NA) among the three treatments was also performed using Fisher's exact test. All of the statistical analyses were carried out in R version 2.15.2 (R Core Team, 2012).

*Eco*RI/*Hpa*II data (0/1 matrices) can be interpreted as DNA methylation variation. To identify the direction of changes in the cytosine methylation, an epigenotype consensus based on scores of the MS-AFLP marker at 0 h was inferred. The observations that were more than or equal to the three deviations among all five replicates were chosen as the starting states of the consensus during the analysis process. DNA methylation variations that were observed in the cold treatments were interpreted as presence (1) or absence (0) according to this epigenotype consensus (S1 Fig).

### Multivariate analysis

To explore and elucidate the patterns of DNA methylationvariations in response to the chilling (4°C) and freezing (-4°C) treatments based on the presence (1) / absence (0) matrix, a non-metric multidimensional scaling (NMDS) analysis with two-dimensional solutions was implemented for DNA methylation variation data (*Eco*RI*/Hpa*II data) [45]. As an ordination technique, the NMDS analysis can assess the dissimilarities of the DNA methylation variations among the treatments (4°C and -4°C for 0, 0.5, 3, 12 and 24 h). In the NMDS analysis, samples can be represented as points apart with distances based on the relative dissimilarity between the samples in a two-dimensional space. The patterns of dissimilarities based on the NMDS analysis were visualized by an appropriate resemblance matrix. All of the NMDS analyses were executed in Past version 1.91 [46].

To visualize and discern the patterns in the differentiation of DNA methylation variations among 23°C, 4°C and -4°C for each of four periods of time (0.5, 3, 12 and 24 h), a principal component analysis (PCA) with Euclidean distance was implemented in the CANOCO for Windows 4.5 software (Biometris, Wageningen, Netherlands). PCA analysis was carried out basing on the presence (1) / absence (0) matrix to generate fewer compound variables (principal components) that characterize the pattern of DNA methylation changes. For each treatment, the randomly selected individual samples were left out for the indicated duration, and the model was recalculated. This recalculation analysis was repeated more than 500 times, the results of which showed that the trends of DNA methylation changes were consistent over the repetitions.

### Chromatin immunoprecipitation assay (ChIP)

ChIP assays were performed as previously described [47]. Chromatin extracts were prepared from *C*. *bungeana* seedlings that were treated with formaldehyde for three periods of cold treatment time (0, 3 and 24 h). The chromatin was sheared to an average length of 200–800 bp by sonication 11 secs. and immunoprecipitated with anti-5-methylcytosine antibody (catalog number MABE146, Merck Millipore). The immunocomplexes were harvested with Protein A agarose and heated with 5M NaCl solution at 65°C for 5 h to release any DNA that was cross-linked to the immunoprecipitated proteins. The amount of each precipitated DNA fragment was determined by real-time PCR with the following cycling conditions: 5 min initial denaturation at 95°C, 40 cycles of 95°C for 15 s, 55°C for 20 s, and 72°C for 20 s, using the primers *CbUGT* fragment (5’-GTGCTTTCGCTAGTTCCTCTAT -3’ and 5’-CGACGATTATAGAGTTTGGCTAGA-3’), *CbADH1* fragment (5’-TGATGGAGGAAGTGGAAGTTG-3’ and 5’-GGTGTGACAGAGAGAAGTGAAG-3’) and *CbPGIP* fragment (5’-CCTCCTCAAGATCAAGAAATCTCTAA-3’ and 5’-CAGTACCAGGAGCAACAGTC-3’). To assess non-specific binding, an immunoprecipitation reaction without antibody was performed as a negative control. Meanwhile, the input was performed as a positive control.

### Quantitative real-time PCR (qPCR)

Total RNA was extracted from control or chilling- or freezing-stressed *C*. *bungean*a seedlings using the plant total RNA extraction kit (Tiangen, Beijing) and treated (20 μg RNA) with 1 U of DNase (TAKARA, Japan). Then, 1 μg of DNase-treated RNA was used to produce the first strand of cDNA using 200 U of M-MLV reverse transcriptase (Promega, USA) and analyzed with Platinum SYBR green qPCR supermix-UDG reagents (Invitrogen). qPCR analysis was performed with three technical repeats for each sample. The relative expression levels of target genes were normalized with the three selected reference genes of *C*. *bungean*a with the Pfaffl method (CBT10872/AT3G60800, CBT28565/AT5G27630 and CBT12464/AT2G28390) and expressed most stably in chilling- and freezing-treated samples [35]. The PCR conditions were 5 min at 95°C followed by 40 cycles of the following: 15 s at 95°C and 1 min at 60°C. The gene-specific primers for real-time PCR analysis were designed using the www.idtdna.com/Primerquest and Primer Premier (version 5.0) software (PREMIER Biosoft). The primer pairs that were used were CBT10872 (5’-CACTTAAAGCAAACGCCAAGTTC-3’ and 5’-GCCGCATTTCATTGCGTTCT-3’), CBT28565 (5’-TCAACCTCCATCTCGGACTCA -3’ and 5’-TGGATACAACGGACGTTACAACA-3’), CBT12464 (5’-TGGGATAAACTGCCCCATTGT-3’ and 5’-AGAAACCAGTCATACGCAAGAATC-3’), *CbADH1* (CBT4440) (5’- GCACAGGAAGCGTTCAGGC-3’ and 5’- GGATGTCAGTTTTGGGTTTGTAGTT-3’), *CbUGT* (CBT18111) (5’- CTTCTCCTTCTGTCTTCGTGTATC-3’ and 5’- GGAACTAGCGAAAGCACTCATA -3’), and *CbPGIP* (EU441203) [33] (5’-GATGCTTCGATGTTGTTTGGA-3’ and 5’-ACTCCCTGTAATCCCGTTGTG-3’).

## Results

### The changes in DNA methylation in response to the cold treatments

**Fig 2:**
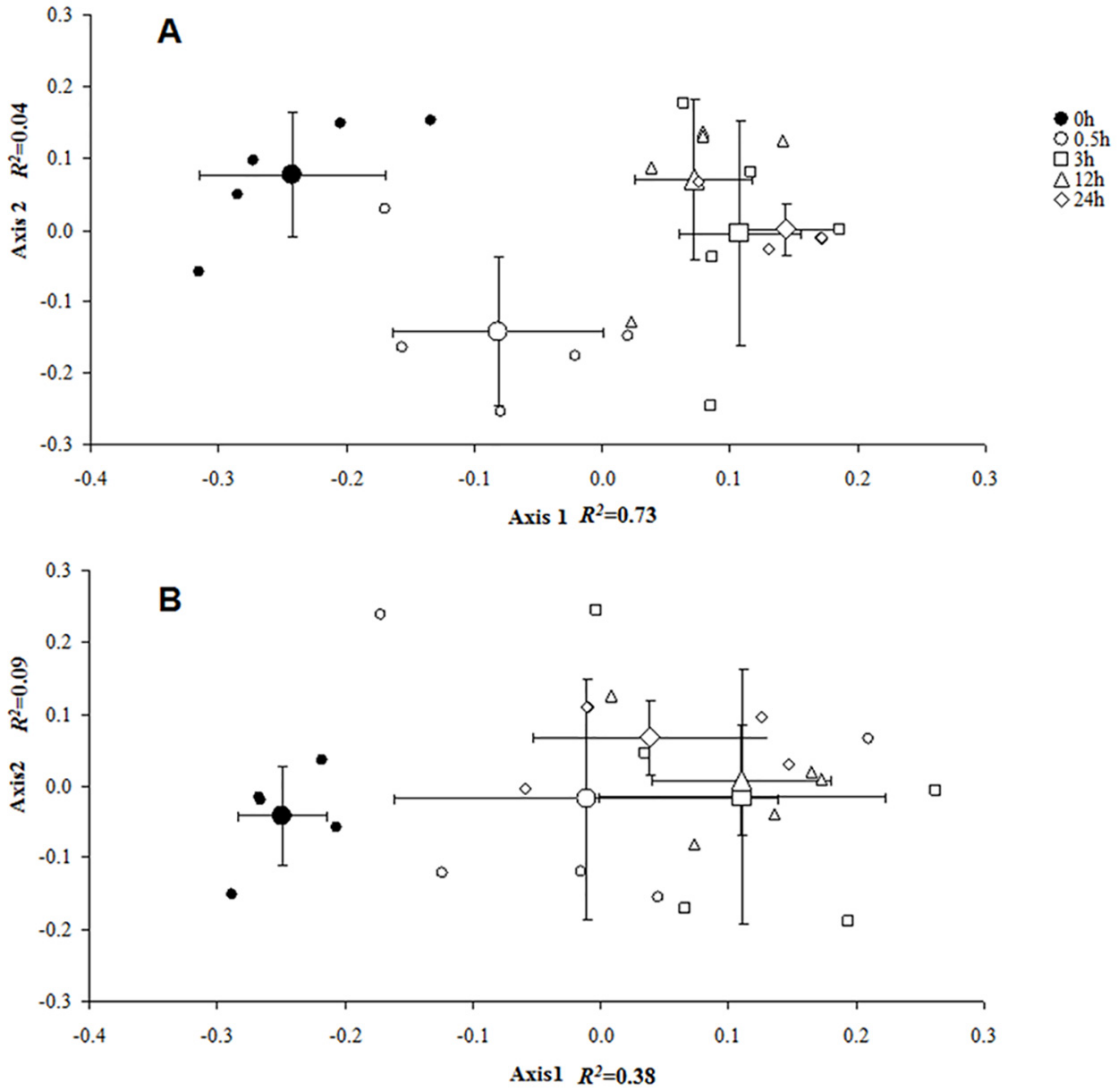
Non-metric multidimensional scaling (NMDS) analysis for variation in methylation epigenotypes. Non-metric multidimensional scaling representing the variation in methylation epigenotypes between samples. The dynamic pattern of epigenetic divergence among the 0, 0.5, 3, 12 and 24 h in 23°C, 4°C and -4°C treatments based on presence (1) ⁄ absence (0) scores of 16 polymorphic methylation-sensitive amplified fragment-length polymorphism (MS-AFLP) markers. The first two components of the NMDS analysis are extracted and plotted against each other; the small symbols are individual plants (n = 5), while the large symbols with the cross indicate the mean ± SD of the treatment group. (A) At 4°C for 0–24 h. (B) At -4°C for 0–24 h. *R* ^2^ values represent the proportion of the variance that is explained by the first two components. Axis 1 explains the majority of the total variation in both (A) and (B).

DNA methylation changes were significantly activated over the period of chilling compared to those at 23°C and marginally significantly activated at -4°C for 0.5 h compared to those at 23°C for 0.5 h (Table 1). Indeed, the modifications in DNA methylation (methylation and demethylation events) largely occurred under the chilling (4°C) and freezing (-4°C) stresses in the other four time periods compared to those at 0 h (Fig 2 and Table 2). For instance, the proportion of methylation changes in the total 80 cases was greater than 30% (for demethylation) and greater than 10% (for methylation) under chilling (4°C), while the proportion was only 7.5% (for demethylation) and 2.5% (for methylation) at 0 h (Table 2). The result was similar for the proportion of methylation changes under freezing (-4°C) compared to those at 0 h. The temporal (0–24 h) dynamic pattern of the epigenetic differentiation also showed a remarkable divergence between the initial 0 h and the cold stresses that were continued for 0.5, 3, 12 and 24 h (Fig 2). Indeed, the patterns of methylation changes were significantly different between 23°C and the cold treatments (4°C or -4°C), based on their comparisons using the *G* test (Table 3).

**Table 1:** Results of the generalized linear model (GLM) testing of the effects of cold treatments on DNA methylation variations of *C*. *bungeana* genome in response to chilling (4°C) and freezing (-4°C) for five periods of time (0–24 h).

	DNA methylation profile		
	*df*	*χ2*	*P* value
Treatments	1	4.943	**0.026** ^1^
Markers	15	175.2	**<0.001**
Time	1	0.031	0.859
23°C/4°C	1	6.808	**0.009**
23°C/0.5 h & 4°C/0.5 h	1	4.245	**0.039**
23°C/3 h & 4°C/3 h	1	5.667	**0.017**
23°C/12 h & 4°C/12 h	1	4.929	**0.026**
23°C/24 h & 4°C/24 h	1	2.508	0.113
23°C/-4°C	1	0.306	0.580
23°C/0.5 h & -4°C/0.5 h	1	3.614	0.0571
23°C/3 h & -4°C/3 h	1	0.228	0.633
23°C/12 h & -4°C/12 h	1	0.233	0.629
23°C/24 h & -4°C/24 h	1	0.026	0.873

In the model, dummy indicator variables are manually constructed for eight cold treatments (at 4°C and -4°C for 0.5, 3, 12 and 24 h). The difference between each of the eight cold treatments and the 23°C (for 0.5, 3, 12 and 24 h) is assessed by comparing the full model with the model from which the respective term is removed using the Generalized likelihood ratio test (GLRT). The effects of markers (different primer pairs) and treatment times are also examined.

^1^, Significant effects (*p*<0.05) are printed in bold, while the number in italic font indicates marginal significance (0.05<*p*<0.1).

**Table 2:** Methylation changes in *C*. *bungeana* subjected to the chilling (4°C) and freezing (-4°C) treatments for five periods of time (0–24 h).

		4°C				-4°C			
		Methylation change		% changed		Methylation change		% changed	
Group	marker× sample^1^	(0>1)^2^	(1>0)^2^	Demethylation	Methylation	(0>1)^2^	(1>0)^2^	Demethylation	Methylation
0 h	80	6	2	7.5	2.5	6	2	7.5	2.5
0.5 h	80	25	9	31.25	11.25	27	11	33.75	13.75
3 h	80	34	15	42.5	18.75	29	22	36.25	27.5
12 h	80	26	9	32.5	11.25	20	19	25	23.75
24 h	80	27	13	33.75	16.25	16	13	20	16.25

In *Eco*RI/*Hpa*II data, the presence (1) / absence (0) scores of 16 MS-AFLP loci at 4°C and -4°C are identified based on the consensus epigenotype at 0 h. At 4°C and -4°C, the changes in DNA methylation are inferred as either demethylation or methylation based on their divergence from the consensus epigenotype (0 h). The percentage of demethylation and methylation in the total 80 cases is also displayed.

^1^, 16 MS-AFLP markers that were scored in each of five replicate plants for a total of 80 cases per group.

^2^, Direction of the methylation change: 0>1, from absent to present; 1>0, from present to absent.

**Table 3:** *G* test for the frequencies of DNA methylation changes (i.e., methylation, demethylation and NA) of *C*. *bungeana* in response to the chilling (4°C) and freezing (-4°C) stresses.

DNA methylation changes			
	*df*	*χ2*	*P* value
**23°C vs. all other treatments**			
Between groups	8	70.924	**<0.001** ^1^
23°C/0.5 h vs. 4°C/0.5 h	1	23.100	**<0.001**
23°C/3 h vs. 4°C/3 h	1	49.562	**<0.001**
23°C/12 h vs. 4°C/12 h	1	24.580	**<0.001**
23°C/24 h vs. 4°C/24 h	1	32.560	**<0.001**
23°C/0.5 h vs. -4°C/0.5 h	1	29.251	**<0.001**
23°C/3 h vs. -4°C/3 h	1	53.864	**<0.001**
23°C/12 h vs. -4°C/12 h	1	30.885	**<0.001**
23°C/24 h vs. -4°C/24 h	1	16.262	**<0.001**
**23°C vs. 4°C & -4°C**			
Between groups	2	51.434	**<0.001**
23°C vs. 4°C	1	47.324	**<0.001**
23°C vs. -4°C	1	46.690	**<0.001**
4°C vs. -4°C	1	0.006	0.937

The top panel shows the comparisons of the frequency of DNA methylation changes between 23°C and each of the eight treatment groups under chilling (4°C) and freezing (-4°C), while the bottom shows the general comparisons among 23°C, 4°C and -4°C. NA means that the status of DNA methylation of the loci is unknown. All of the comparisons were conducted by *G* test.

^1^, Significant effects (p<0.05) are printed in bold.

Moreover, this DNA methylation modification was activated in a very short time as the cold initiated. For instance, the total demethylation and methylation events amounted to 61.25% and 63.75% of the 80 cases within 3 h in response to chilling and freezing, respectively (Table 2). This response resulted in a rapid (within 3 h) divergence of the methylation variations between 0 h and the other time points under chilling and freezing, as visualized and examined by the NMDS analysis (Fig 2). Interestingly, compared to the methylation events, the occurrences of DNA demethylation were more frequent under both chilling and freezing. For instance, the proportion of demethylation events in the total 80 cases reached 42.5% (for chilling) and 36.25% (for freezing) within 3 h as the cold imposed, while the methylation events were comparatively much lower (Table 2). However, the activities of DNA demethylation appeared to attenuate to a certain degree (e.g., down to 20% at 24 h) as the freezing continued (3–24 h).

### Differentiation in DNA methylation variations between chilling and freezing

The NMDS analysis that assessed the dissimilarities in DNA methylation variations at 0, 0.5, 3, 12, and 24 h under the 4°C or -4°C treatment was plotted to visualize the patterns of DNA methylation changes over the period of cold treatments (0–24 h) (Fig 2). The DNA methylation response to chilling (4°C) was slightly slower than that to freezing (-4°C) at the initial stage of the treatment because the DNA methylation differentiation of chilling (4°C) at 0.5 h from 0 h was not as great as in the case of freezing (-4°C) at 0.5 h (Fig 2). However, as the cold stresses continued (after 3 h), the DNA methylation variations under both chilling and freezing showed a dramatic divergence from that of the 0-h groups. Meanwhile, the methylation pattern of chilling (4°C) at 0.5 h was also significantly different from that at subsequent times (at 3, 12 and 24 h), while this pattern was not true for the freezing (Fig 2), suggesting that the 0.5-h time point is significant in methylation regulations under the chilling treatment.

**Fig 3:**
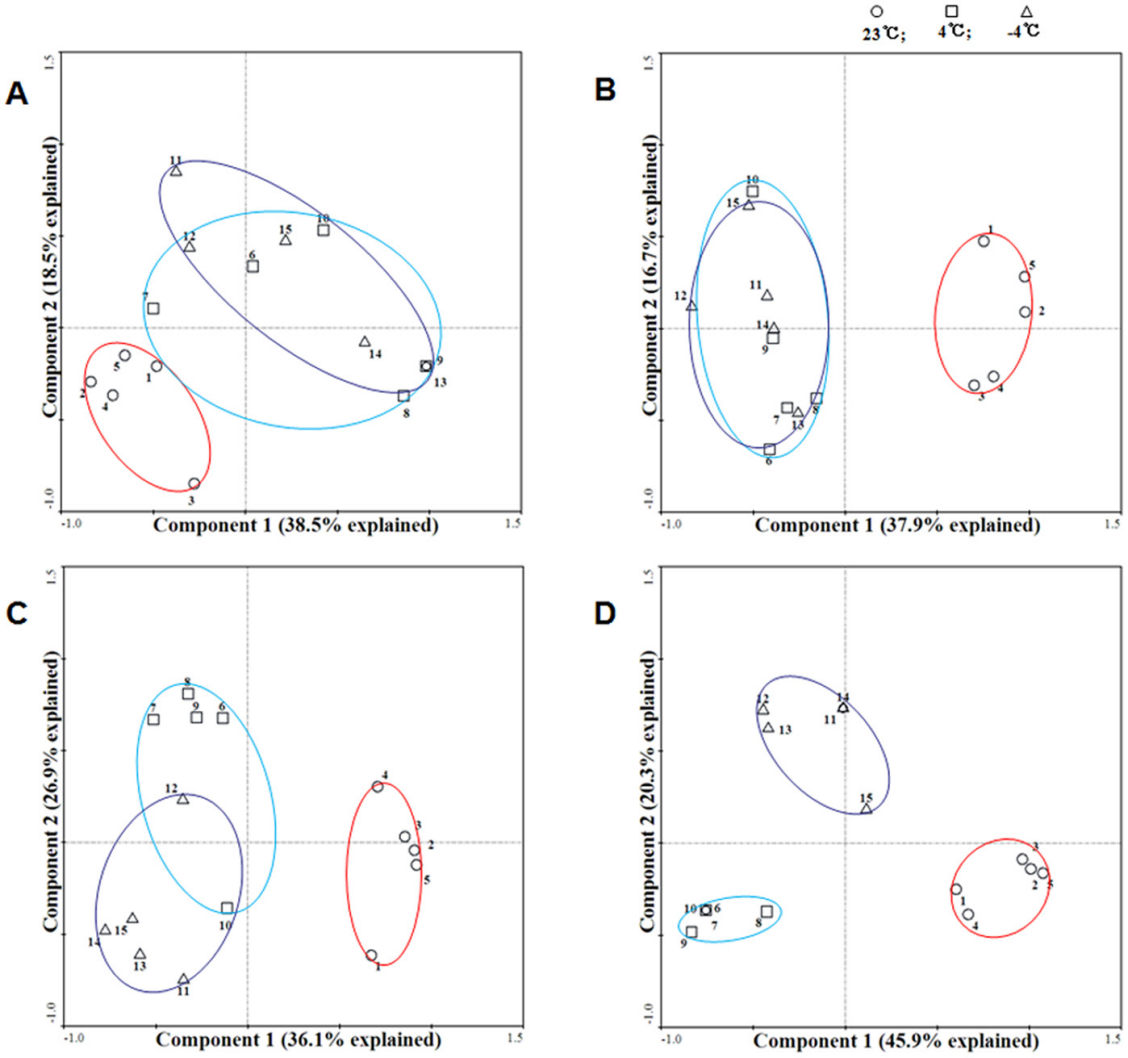
Principal component analysis (PCA) of the different cold treatments. Principal component analysis (PCA) of the comparisons of DNA methylation changes among the 23°C, chilling (4°C) and freezing (-4°C) treatments at each treatment time. (A) 0.5 h, (B) 3 h, (C) 12 h, (D) 24 h. The trends in DNA methylation changes at 23°C, 4°C and -4°C are represented by red circles, cyan circles and purple circles, respectively. Numbers 1–5 represent five individual plants at 23°C, 6–10 represent five individual plants at 4°C, and 11–15 represent five individual plants at -4°C. The percentage of the variance that is explained by each principal component is given in the iconograph near the axes. The first two components together explain greater than 50% of the variations in all of the PCA modes.

To further explore the patterns of DNA methylation differentiation among the 23°C, chilling (4°C) and freezing (-4°C) treatments over time, the PCA was plotted for each of the five periods of time (0, 0.5, 3, 12 and 24 h) (Fig 3). The DNA methylation changes under chilling (4°C, cyan circle) and freezing (-4°C, purple circle) appeared to be fairly converged in the early stage of the cold stresses (Fig 3A and 3B). However, as the cold stresses continued (3–24 h), a reasonable divergence in DNA methylation changes between chilling and freezing gradually emerged (Fig 3C and 3D), which resulted in an obvious separation between the two at the end of the cold treatments (Fig 3D).

Moreover, although the composition of the methylation changes was not significantly different between chilling and freezing (Table 3), the relative proportion of methylation and demethylation was significantly different between the two, based on Fisher's exact test (4°C vs -4°C: χ^2^ = 1.717, *df* = 1, *P* = 0.025). In fact, exposure to freezing caused more frequent DNA methylation, while relatively greater DNA demethylation occurred under chilling (Table 2). Furthermore, as freezing continued (3–24 h), the activities of DNA demethylation appeared to attenuate to a certain degree (e.g., down to 20% at 24 h), while this pattern was not true for the chilling.

### BLAST results of the differentially methylated DNA fragments

Forty-three cold-induced polymorphic DNA methylation bands randomly selected were cloned and sequenced. The sequences of the cloned bands had an average size of 133 bp, ranging from 60 to 226 bp (Table 4), and the results show that these sequences included a CCGG site in one of the termini, verifying the reliability of the MS-AFLP method. BLAST X analysis showed that 34 of the profiles represent active genic sequences. The remaining cloned fragments were related to the transposable, gypsy-like retrotransposons and unknown proteins. Based on the BLAST results (Table 4), some of the cloned fragments were homologous to genes encoding UDP-glucosyltransferase, xylose isomerase, nucleoside triphosphate hydrolases, plant telomeric DNA repeat-binding protein, RUB1-activating enzyme, polygalacturonase-inhibiting protein, and FAD-binding Berberine family protein, and one was homologous to the cDNA sequences involved in the polysaccharide biosynthetic process. In contrast, four proteins were highly homologous to mitochondrial glyoxalase, alcohol dehydrogenase, NagB/RpiA/CoA transferase-like superfamily protein, and disease-resistance protein.

**Table 4:** Isolation and directional sequencing of MS-AFLP fragments. The homologs of *Arabidopsis* genes were presented for functional description.

Sequence (primer and position)	Length (bp)	Homologous sequence	*E* value	Alternation of DNA methylation pattern
E32H39_2–1	101	Transposable element gene, AT2G04600	0.018	Methylation
E36H41_2–2	120	Encodes a protein that specifically binds plant Telomeric DNA repeats, AT5G13820	0.39	Demethylation
E36H41_4–3	184	UDP-glucosyltransferase 75B2, AT1G05560	0.87	Demethylation
E37H39_3–1	95	Regulator of Vps4 activity in the MVB pathway protein, AT4G32350	0.91	Methylation
E38H40_2–2	226	Xylose isomerase family protein, AT5G57655	0.42	Demethylation
E45H39_2–3	173	Encodes a mitochondrial glyoxalase 2, AT2G31350	3e-13	Demethylation
E45H39_5–1	198	P-loop containing nucleoside triphosphate hydrolases superfamily protein, AT5G37150	0.92	Demethylation
E38H40_5–2	156	Catalyzes the reduction of acetaldehyde using NADH as reductant. Alcohol dehydrogenase (NAD) activity, AT1G77120	2e-20	Demethylation
E32-H39_1–1 & E32-H39_2–3	60	Transposable element gene, AT1G36406	0.57	Methylation
E45-H39_6–3	188	Ulp1 protease family, AT4G07580	3.5	Demethylation
E32-H39_1–2	125	Unknown protein, AT1G25422	4e-06	Demethylation
E32-H39_2–2	90	Transducin/WD40 repeat-like superfamily protein, AT2G16405	0.96	Methylation
E36-H41_1–1	195	Unique among Arabidopsis Arm-repeat proteins, AT2G44900	0.079	Demethylation
E36-H41_1–2 & E36-H41_2–2 & E36-H41_2–3	85&123&121	Encodes a protein that specifically binds plant telomeric DNA repeats, AT5G13820	0.089&0.85&0.76	Methylation
E36-H41_3–1 & E36-H41_3–2	206&208	NagB/RpiA/CoA transferase-like superfamily protein, AT3G07300	3e-13	Demethylation
E36-H41_4–1	171	Unknown protein, AT5G14150	3.3	Demethylation
E36-H41_4–3	184	Polysaccharide biosynthetic process, AT3G57220	0.87	Demethylation
E37-H39_1–1	116	RUB1 activating enzyme, AT1G05180	0.98	Demethylation
E37-H39_1–2	114	ABC-2 and Plant PDR ABC-type transporter family protein, AT4G15233	3.8	Methylation
E37-H39_2–1	123	Mutator-like transposase family, AT3G31909	0.25	Demethylation
E37-H39_2–2	122	Unknown protein, AT5G61412	0.25	Demethylation
E37-H39_3–1	195	Regulator of Vps4 activity in the MVB pathway protein, AT4G32350	0.91	Methylation
E37-H39_3–2	193	Protein kinase superfamily protein, AT5G12090	0.90	Methylation
E38-H40_1–1	160	Gypsy-like retrotransposon family (Athila), AT5G33381	1e-06	Methylation
E38-H40_1–2	158	Gypsy-like retrotransposon family, AT5G29380	5e-09	Methylation
E38-H40_1–3	105	Disease resistance protein, AT5G41750	4e-06	Demethylation
E38-H40_2–1	127	Xylose isomerase family protein, AT5G57655	4.0	Demethylation
E45-H39_1–3	197	Unknown protein, AT4G17840	1e-15	Demethylation
E45-H39_2–1	170	Gypsy-like retrotransposon family, AT4G06620	8e-05	Demethylation
E45-H39_2–2	170	Encodes a mitochondrial glyoxalase 2, AT2G31350	2e-20	Demethylation
E45-H39_3–1	145	Encodes a protein containing ankyrin and DHHC-CRD domain, AT5G20350	4.2	Demethylation
E45-H39_3–2	146	F-box family protein, AT1G67130	0.27	Demethylation
E45-H39_3–3	142	ENTH/ANTH/VHS superfamily protein, AT1G03050	0.27	Demethylation
E45-H39_4–1	129	Unknown protein, AT5G20100	4.0	Demethylation
E45-H39_4–2	135	Unknown protein, AT5G37390	1e-15	Demethylation
E45-H39_5–1	98	P-loop containing nucleoside triphosphate hydrolases superfamily protein, AT5G37150	0.92	Demethylation
E45-H39_6–1	93	Gypsy-like retrotransposon family, AT5G34851	2e-04	Methylation
E45-H39_6–2	92	FAD-binding Berberine family protein, AT2G34810	3.6	Methylation
E45-H41_3–3	166	Polygalacturonase-inhibiting protein, AT5G06860	0.023	Demethylation

Methylation occurs if bands only appear in 0h rather than any other time of the cold treatment (0.5, 3, 12, 24h). However, demethylation occurs if the opposite is true.

### 5-Methylcytosine level analysis and qPCR verification

In order to validate the pattern of methylation change at different cold temperatures, *CbADH1*, *CbUGT* and *CbPGIP* were selected from random sequencings to measure the level of binding with 5-methylcytosine and the level of expression under chilling (4°C) and freezing (-4°C). A chromatin immunoprecipitation (ChIP) assay was used to analyze whether the specific expression of interesting genes is accompanied by changes in the 5-methylcytosine state.

**Fig 4:**
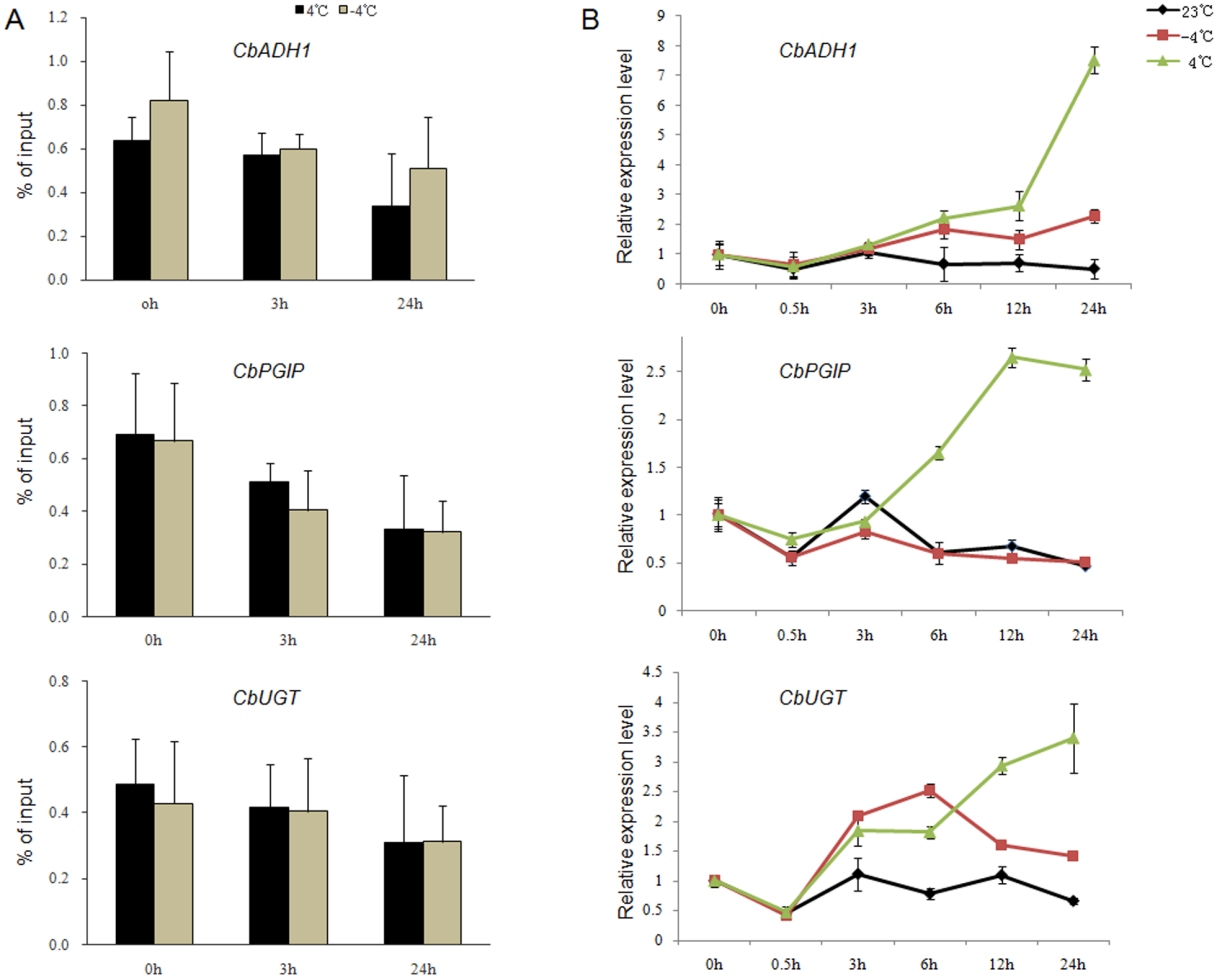
5-Methylcytosine-binding level assays and the expression analysis of three target genes. (A) The levels of *CbADH1*, *CbUGT* and *CbPGIP* bound to 5-methylcytosine in chilling (4°C) and freezing (*-*4°C) treatments. (B) The expression level of these three genes at 23*°C*, chilling (*4°C*) and freezing (*-*4°C).

As shown in Fig 4, the 5-methylcytosine level of *CbADH1*, *CbUGT* and *CbPGIP* similarly decreased during chilling and freezing (Fig 4A). The 5-methylcytosine level of *CbADH1* decreased approximately 2-fold from 0 h to 24 h during the chilling and freezing treatments. However, the level of 5-methylcytosine binding at *CbUGT* and the change degree were relatively small. Whether the decrease in DNA methylation occupancy corresponded to the expression level of genes was determined by the mRNA assays of *CbADH1*, *CbUGT* and *CbPGIP* at different times during chilling and freezing (Fig 4B). The results showed that the expression of three selected genes was significantly upregulated during chilling; among these genes, the expression of *CbADH1* increased 8-fold in the chilling treatment. Except *CbPGIP*, the expression of the other two genes under freezing also increased.

## Discussion

Plants respond to environmental stresses by differentially regulating genome-wide gene expression to adjust physiological activity [48]. Epigenetic mechanisms, such as DNA methylation/demethylation, play an important role in environmentally induced phenotypic and physiological variation [49, 50]. Transcriptome profiling and molecular genetics have analyzed the stress regulatory network of *C*. *bungeana*, a subnival alpine plant with inherited cold tolerance [33–37, 39, 40], but little is known about the direct effects of low temperature on epigenetic regulation. Using a combination of the MS-AFLP technique and integrated statistical analyses, we found an interesting variation in DNA methylation of *C*. *bungeana* in that different patterns appeared in the late response (after 12 h) to the chilling and freezing treatments. In addition, Forty-three cold-induced polymorphic fragments were randomly selected and further analyzed. Some of these sequences are involved in anaerobic metabolism and carbohydrate metabolism, coinciding with a prominent role of central carbohydrate metabolism in cold protection as suggested by the metabolic study [51]. Furthermore, the level of 5-methylcytosine binding at *CbGUT*, *CbADH1* and *CbPGIP* and their mRNA expression were tested by chromatin immunoprecipitation assays (ChIP) and qPCR analysis, and the results showed that the level of 5-methylcytosine is reduced during cold treatments and that the potential divergence of the DNA methylation pattern between chilling and freezing does exist with the continuation of cold stress. Our study finds the variation of DNA methylation pattern for alpine plant cold tolerance. We propose that DNA methylation/demethylation can potentially serve as a rapid and flexible adaptive mechanism to deal with intricate cold stress to protect *C*. *bungeana* from freezing damage in its habitats.

In our study, *C*. *bungeana* was investigated for special cold tolerance. This species is an alpine plant that naturally grows and survives in Asian alpine regions (www.efloras.org), where cold stress is often intricate because the air temperature frequently fluctuates from chilling to freezing within a day during the growing seasons of *C*. *bungeana*. In this case, epigenetic regulation may serve as a relatively fast and flexible mechanism in the adaptation of *C*. *bungeana* to intricate cold stresses. More importantly, this epigenetic variation as special soft inheritance may have contributed to the adaptive evolution of *C*. *bungeana* to cold stresses in its natural habitats, independent of evolutionary changes merely based on the genetic variations [52–55]. Therefore, *C*. *bungeana* is an ideal natural system to study the role of the epigenetic regulation in the adaption of alpine plant species to extreme and intricate living conditions. Methylation-sensitive amplified fragment-length polymorphism (MS-AFLP) is a simple and reliable technique that has been frequently used to analyze the genomic DNA methylation of non-model organisms because this methodology does not depend on the availability of the genome sequence [31]. DNA methylation patterns are usually analyzed by MSAP combining *Eco*RI/*Hpa*II with *Eco*RI/*Msp*I digestion, but here, only *Eco*RI/*Hpa*II was used. In Catarina’s study, because hemimethylated loci are not inherited over generations, *Msp*I sensitivity to non-symmetrical methylated sites was bypassed, and the *Eco*RI/*Msp*I data analysis was extrapolated as the genetic profile. In contrast, *Eco*RI/*Hpa*II profiles were considered as epigenetic structures (represented by the CpG-methylation sensitivity of *Hpa*II) [30]. For example, *Hpa*II/*Mse*I was used in the study of stress-induced DNA methylation changes and their heritability in asexual dandelions [32]. Thus, it is feasible and reliable that *Eco*RI/*Hpa*II arises as the result of variation among samples in the DNA methylation status of marker loci.

Indeed, our results show that the modifications in DNA methylation (methylation and demethylation) of *C*. *bungeana* largely occur over a period of both chilling and freezing stresses. Through the random sequencing of cold-induced polymorphic DNA methylation bands, some of these genes were found to be involved in saccharide biosynthetic and metabolism process, energy supply and transcriptional regulation. Therefore, changes in DNA methylation are involved in the transcriptional regulatory networks of the expression of some known genes in anaerobic metabolism (e.g., alcohol dehydrogenase) and carbohydrate metabolism (e.g., UDP-glucosyltransferase, xylose isomerase, and the cDNA sequences involved in the polysaccharide biosynthetic process) of *C*. *bungeana* in response to chilling and freezing. Among these genes, the expression of *CbUGT* is coincident with the top 50 up-regulated unigenes of *C*. *bungeana* by chilling stress in transcriptome analysis [35], in agreement with a role of UGT in modulating *Arabidopsis* drought and salt stress tolerance [56]. The expression of *CbPGIP* agrees with previous research [33], indicating that changes of *CbPGIP* expression are regulated by DNA methylation/demethylation, in agreement with the up-regulation of *Arabidopsis PGIP* by demethylation [57]. In addition, changes in the expression of *CbADH* were found in this study. Alcohol dehydrogenase (ADH) widely exists in living organisms, it plays extremely important roles in growth, development and stress resistance in plants and can be induced by dehydration and cold [58, 59]. It is interesting to explore the relationship between the activity of CbADH and epigenetic regulation.

Cold-induced DNA methylation changes consist of methylation and demethylation, which are usually involved in distinct regulatory processes by activating or inhibiting the transcriptional activities of different genes [20]. We found that the occurrences of demethylation were in general more frequent than methylation events under the cold stresses. In agreement with our results, a couple of recent studies reported that the frequency of DNA demethylation increased under cold treatments [60, 61]. However, DNA methylation mostly occurred in transposons and retrotransposons (Table 4), probably related to maintaining the cell stability under stress.

In conclusion, our study showed that exposure to the cold induced rapid and large-scale DNA methylation changes in *C*. *bungeana*, and this epigenetic response was different between chilling (4°C) and freezing (-4°C). Previous studies have indicated that cellular and molecular processes are involved in the adaptation of *C*. *bungeana* to cold stresses [38, 62–66]. This is the first study to address the potential role of epigenetic variations (DNA methylation/demethylation) in the adaption of *C*. *bungeana* to the fluctuating chilling and freezing in the alpine freeze-thaw tundra in the Tianshan mountain. Based on the results, we conclude that epigenetic variation can serve as a rapid and flexible regulatory mechanism for *C*. *bungeana* to adapt to intricate cold stresses. Our findings can also contribute to understanding how alpine plant species cope with extreme, dynamic and intricate cold in their habitats, especially under the context of global climate change [67].

## Supporting Information

S1 Fig MS-AFLP presence/absence polymorphisms detected in the experiment (*Eco*RI/*Hpa*II dataset). (DOCX)

S2 Fig Part of MS-AFLP fingerprints. (DOCX)

S1 Table Adapters and primers of MS-AFLP analysis. (DOCX)
