# Supplementary material for: Chilling- and Freezing- Induced Alterations in Cytosine Methylation and Its Association with the Cold Tolerance of an Alpine Subnival Plant, Chorispora bungeana
Source: PLoS One. 2015 Aug 13;10(8):e0135485. doi: 10.1371/journal.pone.0135485 (PMC4535906; doi:10.1371/journal.pone.0135485)
Supplement: S1 Fig — (DOCX) [file pone.0135485.s001.docx]

**S1 Figure**. **MS-AFLP presence/absence polymorphisms detected in the experiment (*Eco*RI/*Hpa*II dataset).**


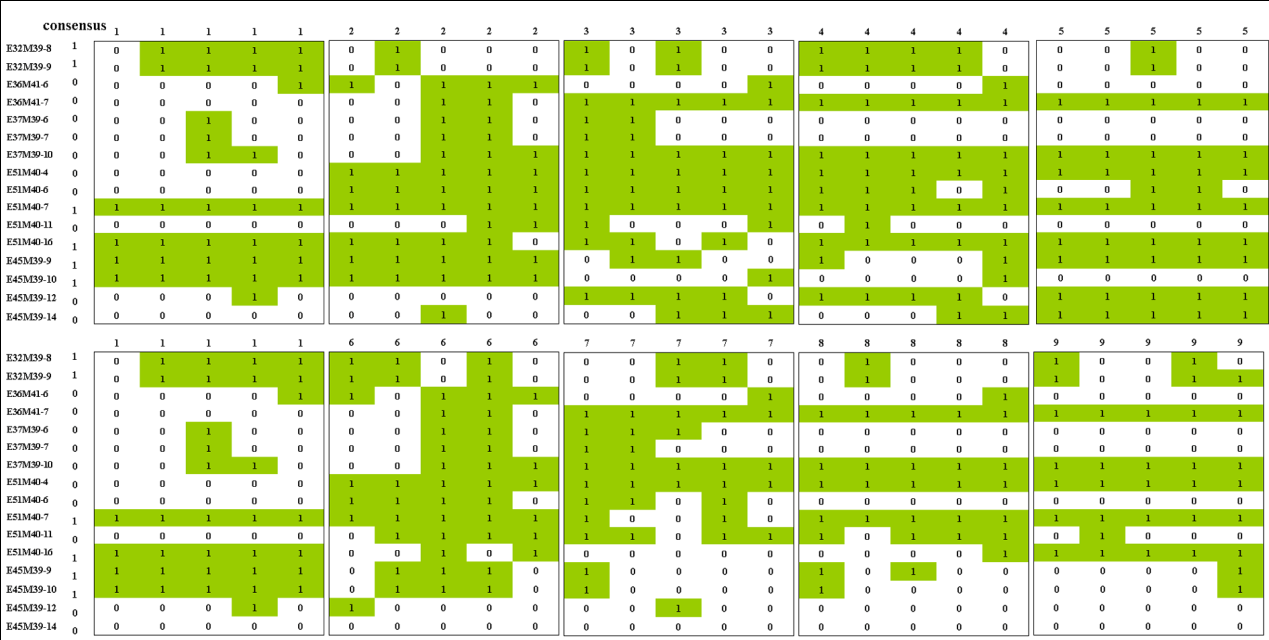


Supporting information Fig. 4

80×160mm (Tiff at 300DPI)

**Figure S1**. ***Eco*RI/*Hpa*II dataset, ie., MSAP presence/absence polymorphisms detected in the experiment.**

The 16 primer markers are in rows, and individual plants are in columns grouped by the treatments. On the top panel, the headings 1-5 refer to the treatments at 4°C in 0, 0.5, 3, 12 and 24 h; on the bottom panel, the headings 6-9 refer to the treatments at -4°C in 0, 0.5, 3, 12 and 24 h. The consensus epigenotype is indicated on the left edge of panels, which is based on the observation that selected more than or equal to three deviating among five replicates in the 0h group.
