# Supplementary material for: Chilling- and Freezing- Induced Alterations in Cytosine Methylation and Its Association with the Cold Tolerance of an Alpine Subnival Plant, Chorispora bungeana
Source: PLoS One. 2015 Aug 13;10(8):e0135485. doi: 10.1371/journal.pone.0135485 (PMC4535906; doi:10.1371/journal.pone.0135485)
Supplement: S2 Fig — (DOCX) [file pone.0135485.s002.docx]

**S2 Figure. Part of MS-AFLP fingerprints.**

**
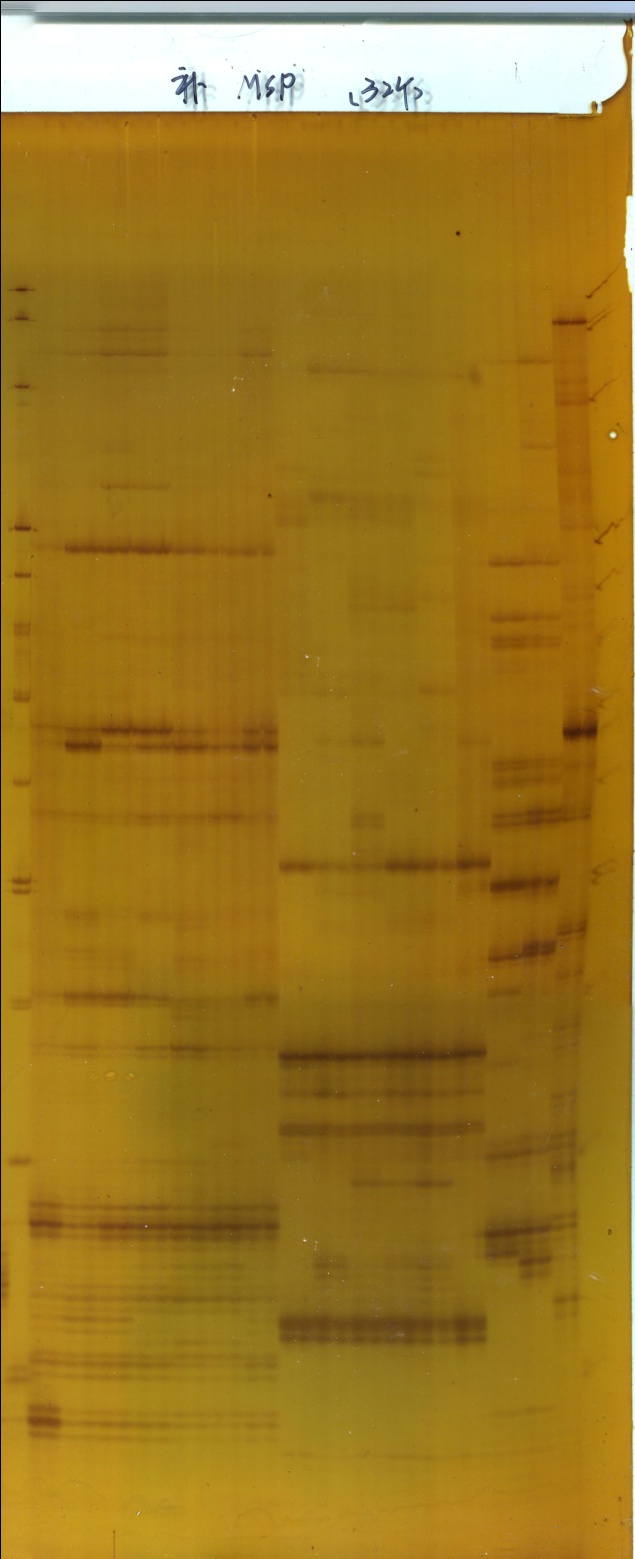
**

**S2 Figure. Part of MS-AFLP fingerprints.**

Lane M: 1 KB Ladder; Lane 2 to 9: E36/Msp41; Lane 10 to 16: E38/Msp40; Lane 17 and 18: E32/Msp39; Lane 20: E37/Msp39.
