# Supplementary material for: Chilling- and Freezing- Induced Alterations in Cytosine Methylation and Its Association with the Cold Tolerance of an Alpine Subnival Plant, Chorispora bungeana
Source: PLoS One. 2015 Aug 13;10(8):e0135485. doi: 10.1371/journal.pone.0135485 (PMC4535906; doi:10.1371/journal.pone.0135485)
Supplement: S1 Table — (DOCX) [file pone.0135485.s003.docx]

S1 Table. Adapters and primers of MS-AFLP analysis

| Adapters and primers | Sequence (5'-3') | Primers | Sequence (5'-3') |
| --- | --- | --- | --- |
| EcoRI adapters1 | CTCGTAGACTGCGTACC | E37 | GACTGCGTACCAATTCACC |
| EcoRI adapters1 | AATTGGTACGCAGTCTAC | E38 | GACTGCGTACCAATTCACT |
| HM adapters1 | GACGATGAGTCCTGAG | E45 | GACTGCGTACCAATTCATG |
| HM adapters2 | CGCTCAGGACTCAT | E51 | GACTGCGTACCAATTCCAT |
| E00 | GACTGCGTACCAATTC | Msp39 | GATGAGTCCTGAGCGGAGA |
| MSP00 | GATGAGTCCTGAGCGG | Msp40 | GATGAGTCCTGAGCGGAGC |
| E32 | GACTGCGTACCAATTCAAC | Msp41 | GATGAGTCCTGAGCGGAGG |
